# Presumption of Survivorship

**Published:** 1843-05

**Authors:** 


### Selections from American and Foreign Journals.

---

*Presumption of Survivorship.*—By Dr. Krugelstein. The cases in which the presumption of survivorship may arise, are the following. 1. When mother and child both die during delivery. 2. When many persons perish at the same time, as under the ruins of a falling building, or in a sandpit, or by an earthquake, or by the fumes of charcoal; or when many perish at the same time by shipwreck, or in drowning, when hurled together into an abyss; destroyed in a conflagration; simultaneously poisoned; dying at the same time from wounds; from hunger or from cold.

A. On the presumption of survivorship, when the mother and child die during delivery, two cases may arise. 1. The mother dies during delivery, without bringing forth the child. Here there may be a legal question, whether the right of inheritance of the child, though living, but not born, could be transferred to a third person.

2. The mother dies during delivery, and after her death, the child is born and found dead, no one having noticed its delivery.

In the first instance, it must be previously ascertained what was the cause of the death of the mother, and what the presentation of the child. If the latter is natural, and the parts exhibit mechanical impediments to delivery, and the mother died suddenly of a nervous affection, then the presumption is in favor of the child surviving. Opposed to this, in the opinion of Jörg, is the case cited by him, of a robust and remarkably healthy peasant woman, who for several weeks previous to the full time, suffered under *eclampsia gravidarum*, and during one of these convulsions, without its being noticed by any one, brought forth a dead child, with the placenta. He remarks, however, that *eclampsia* usually attacks only healthy, muscular, and especially full-blooded women; and that the probability of the death of the fœtus, either before or during delivery, is increased according to the length of time that the mother has been suffering under the disease.

But if the position of the child be unnatural, and especially if the funis has protruded, we may assume (and above all, if the pains have been violent) that the mother died after the child.

In the case of a child full grown, and capable of living, born after the death of the mother, the proof by examining the lungs is conclusive as to its life. But if we find on it no signs of maturity, or of intra-uterine life, the presumption is in favor of the mother surviving, and particularly if there be marks of putrefaction in the fœtus.

B. When many persons are destroyed at the same time, the first inquiry is as to the cause of death, whether by suffocation, hunger, thirst, or wounds.

In cases of suffocation, we must notice the age, condition of body, sex, and the position of the dead. In reference to age, children and young persons survive old ones. Thus, in the earthquake at Calabria, a man and his wife, with their child, were entombed by a falling house. On being dug out, the parents were found dead, and the girl alive. The nearer the individual is to the age of childhood, the less is the necessity for respiration; and hence, persons of manly age, and if not asthmatic, but of sound lungs, survive the aged.

In reference to the condition of the body, the most important point is the state of the lungs. A person with sound lungs will easily outlive another, whose lungs are indurated or suppurating, since an unfrequent, but perfect inspiration suffices to preserve for a time, the functions of the lungs, while a short and confined one does not convey sufficient air to them. In reference to the situation, we assume, that those have died last, to whose lungs the access of air was in some degree possible.

But the possibility of obtaining respirable air, depends often on very different and apparently opposite circumstances. In a conflagration which broke out in this city (————————) in 1808, two persons by the ruins falling down before the door of the cellar, were enclosed therein. One was an old man of seventy, and asthmatic, the other a very healthy person, aged about forty. The latter stood upright, and was near suffocating, in consequence of a fine smoke, which penetrated into the cellar and filled the upper half thereof; the old man, on the contrary, who had sat down on the floor, experienced nothing af this inconvenience. A man, who with his daughters and his mother aged seventy, retired into a cellar, on account of fire, was suffocated with his children; while his aged mother was taken out the next day, half dead, gasping for air, but recovered and lived some years thereafter.

The nature of the masses which cause the entombment produce various effects, while the wounds depend on the masses causing them, the height from which they fall, and also the position in which the injured parts are. If large and heavy masses, ruins of walls, rocks, beams, stones, &c., fall upon the body, although the external wound may not appear severe, yet on dissection, we shall find the large blood-vessels, and the heart itself lacerated, and as the extravasated blood stains the parts, it is very difficult to decide whether the living man or his recent corpse was injured. Yet in the last case, the countenance is composed, or certainly not so distorted, as when a man has died in great terror and pain, and receiving very severe wounds.

Masses which do not cohere together, as earth, sand, rubbish, &c., press together the injured parts heavily, without generally breaking them. They also separate the extremities from the trunk, while they press on the space between, as, for example, between the arms and body. In the case of a female overwhelmed in a sand-pit, I found the body so much compressed, that it had scarcely half its natural thickness; besides this, however, there was no external injury observable. Occasionally portions of the falling masses press upon the openings of the softer parts, as in the eyelids, mouth, &c. The abdomen will be pressed together, and the contents of the intestines and bladder forced out, and sometimes even the contents of the stomach will be driven out through the mouth and nose.

Should we find such persons in various situations and positions, and in which it is evident that the deceased could not have been placed after death, as with the extremities drawn from the body, the arms upraised, or resting like the feet on the ground, or if we find under the finger nails, sand, &c., as if the sufferer had endeavored to extricate himself, or if some of these foreign substances are seen in the mouth and windpipe, it is beyond a question that such an one must have survived another, on whom these marks are not found.

It was remarked of those who were entombed by the earthquake in Calabria, that the last position at the moment of death, of males, was an exertion of all muscles, in apparent struggling; while that of the female sex exhibited marks of the wildest despair. The latter, particularly, had their hands clasped above their heads. But when there were any children with the mother, she evidently thought only of protecting them, and with her own body endeavored to ward off the danger. The father, on the contrary, seized his child and then opposed himself to it. Thus at Polestina, a mother with her two children, one a boy of three years old and the other an infant of seven months, was found. The infant was pressed to her breast, while her body was bent over the other, so as to oppose her back to the falling ruins. She held both children firmly inclosed within her arms, and in this position was found under the ruins.

The following case was submitted for the opinion of Pyl. Two married persons had gone to bed in good health. The woman had, however, been for some months feeble, and suffered frequently from faintness and headache. In the stove was found some charred oak wood, there was also an extinguished lamp in the room, but no smoke or vapour could be discovered, although the ventilator was closed.

Both were found dead, and as was supposed, from the fumes of charcoal. The relatives raised a suit about the estate. Those of the husband contended that he must have survived, as he was of a robust constitution, and thus resisted the deleterious effects of the charcoal fumes longer than his feeble wife. Besides, his body was found warm in bed, while hers was cold. On the other hand, the friends of the wife objected, that it was not by any means certain that in all cases the weaker would yield sooner than the stronger. The body of the husband was probably covered with the bed-clothes, and thus preserved its heat, whilst it was ascertained that that of the female was naked. They relied, however, principally on the fact that the wife was only 20 years old and the husband 21, and hence she, as the younger, must have survived.

The following were the appearances externally and on dissection. Both were found in the bed, the woman with folded hands, and body stretched out upon her back, while from the mouth issued a very fetid and rather bloody froth; and from the parts of generation light red blood, which also stained the bed-clothes. The husband lay near her also stretched out, but the upper extremities were stiffer, and his fingers drawn together convulsively. A blackish froth issued from his mouth. All the depending parts of the body in each was of a black and blue color, but in neither could any mark of wound or injury be discovered. The color of putrefaction was present, but the outer skin was firm.

On the dissection of the female, the abdomen was found greatly distended; the intestines exhibited some livid spots; the stomach was much enlarged, its upper part inflamed and the blood-vessels swollen, while the inner surface contained large black spots of the size of a dollar, on which the villous coat was abraded. The impregnated uterus was inflamed on its surface. The lungs were pale and collapsed, and in them and in the heart the blood was small in quantity, but frothy, black, and fluid. The blood-vessels of the brain were greatly distended with black, thin blood.

In the husband the appearances were similar, but more marked. There was more serum in the abdomen, and the stomach was more inflamed. The liver dark colored, contained much fluid, frothy blood. The lungs were more swollen, here and there discolored, and the vessels full of blood. The heart contained more blood than in the female, but the condition of the brain was very similar.

The opinion of Pyl is to the following effect: While all cases of this description are extremely perplexing, and it is indeed impossible to arrive at a conclusion with absolute certainty, the difficulty is here increased by the fact that the period of death in these persons is uncertain, and that both, when found, were already stiff and cold. Their appearance was that of ordinary sleep, neither their countenance nor their limbs were distorted, and it is hence highly probable that both were deprived of life at the same instant. They were apparently suffocated during sleep, or otherwise we should have noticed some indication of an attempt to restore themselves. A few minutes at the most could have intervened between their deaths, and from the examination, it would appear that the effects on the husband were the most rapid and decisive, as shown by the state of the heart and lungs. Still, as the woman was the weakest, and was subject to faintings, she may have died first. In fine, it is impossible to answer the question in a positive manner.

Our author objects to this indecision, and remarks that Pyl should have made a distinction between the various effects of the fumes of charcoal, as they either first attack the brain and cause faintness and apoplexy, or else suffocation (choking rheum). Now, on the body of the wife, no marks of the latter were discovered, whilst they were on that of the husband, and the probability therefore is that the wife died first.

Metzger has also noticed this case, and declares that he would have decided for the earlier death of the wife.—*Amer. Journ. Med. Scien.*, from *Wildberg's Jahrbuch der Gesammtén Staatsarzneikunde.*

(*To be continued.*)

